# Fufang-Zhenzhu-Tiaozhi Capsule reduces restenosis via the downregulation of NF-kappaB and inflammatory factors in rabbits

**DOI:** 10.1186/s12944-018-0921-3

**Published:** 2018-11-30

**Authors:** Rendan Zhang, Tudi Li, Jiao Guo, Yanqun Zhao, Yuhong Liu, Yusi Yao, Zhihuan Zeng

**Affiliations:** 1 Department of Cardiology, Boai Hospital of Zhongshan, Zhongshan, 528403 China; 2 Department of Cardiology, The First Affiliated Hospital of Guangdong Pharmaceutical University, Guangzhou, 510080 Guangdong China

**Keywords:** FTZ, NF-κB, Animal model, Restenosis, Inflammation

## Abstract

**Background:**

To investigate the effects of a Chinese herbal medicine Fufang-Zhenzhu Tiaozhi Capsule (FTZ) on restenosis and elucidate the mechanism of action.

**Methods:**

A restenosis model was established by balloon rubbing the endothelium of the abdominal aorta followed by high fat diet. Rabbits were divided into blank control group, restenosis group, FTZ group (0.66 mg/kg/day), atorvastatin group (5 mg/kg/day) and FTZ + atorvastatin group (*n* = 8). Vascular stenosis was analyzed by X-ray. Serum levels of chemokines and cytokines interleukin-1 (IL-1), interleukin-6 (IL-6), interleukin-8 (IL-8), interleukin-12 (IL-12), C-reactive protein (CRP), tumor necrosis factor-alpha (TNF-α), monocyte chemoattractant protein-1 (MCP-1), and intercellular adhesion molecule-1 (ICAM-1) were measured by ELISA. The levels of NF-κB, IκB-α, P-IκBα, IKK-α, and P-IKKα/β from injured abdominal arteries were detected by Western blotting.

**Results:**

Restenosis was induced successfully via abdominal artery balloon injuries and high fat diet. Restenosis was significantly decreased in FTZ group compared with restenosis group (*P < 0.05*). FTZ group had markedly reduced serum lipid levels (*P < 0.05*). In addition, the levels of TNF-α, IL-1, IL-6, IL-8, IL-12, ICAM-1 and MCP-1 decreased by FTZ treatment (*P < 0.05*). The expression of NF-κB in the atherosclerotic lesions was significantly attenuated in FTZ group (*P < 0.05*).

**Conclusion:**

FTZ could reduce restenosis via reducing NF-κB activity and inflammatory factor expression within the atherosclerotic lesion in a rabbit restenosis model. FTZ may be a new therapeutic agent for restenosis.

## Introduction

Percutaneous coronary intervention (PCI) has been widely used in the treatment of coronary artery disease. Despite the decrease of restenosis in the era of stenting, post-operative arterial restenosis remains a problem [1]. Restenosis is characterized by intimal hyperplasia, smooth muscle cell proliferation, and vascular renarrowing, and the rate of restenosis following PCI is higher in patients with hyperlipidemia, and the recurrence time of angina is highly related to the history of hyperlipidemia [2–4]. Recent studies have demonstrated that obese adipose tissue accelerates the progression of inflammation and promotes the development of atherosclerosis [5]. Lipid lowering therapy, especially targeting low-density lipoprotein cholesterol (LDL-C) < 70 mg/dL, can alter the pattern of restenosis after PCI [6].

According to current guidelines, lipid lowering agents including statins should be prescribed in subjects with coronary artery disease after PCI [7]. However, these agents have side effects. Fufang-Zhenzhu Tiaozhi Capsule (FTZ) is a Chinese herbal medicine prescription including Rhizoma Coptidis, Radix Salvia Miltiorrhiza, Radix Notoginseng, Fructus Ligustri Lucidi, Herba Cirsii Jeponici, Cortex Eucommiae, Fructus Citri Sarcodactylis and Radix Atractylodes Macrocephala, and has been used clinically to reduce serum levels of total cholesterol (TC), triglycerides (TG) and LDL-C, while increase high-density lipoprotein cholesterol (HDL-C) in subjects with dyslipidemia [8]. FTZ exhibits a variety of pharmacological properties, such as alleviating inflammation, regulating blood coagulation, improving endothelial function, and inhibiting the formation and development of atherosclerosis plaque [9]. However, whether FTZ has the efficacy to reduce restenosis and the underlying mechanisms are unknown. The aim of this study was to investigate the effects of FTZ on restenosis and explore whether it is mediated by the regulation of inflammation.

## Materials and methods

### Drugs

Herbs in FTZ [composed of Ligustri Lucidi Fructus (LLF), Salviae Miltiorrhizae Radix et Rhizoma (SMR), Coptidis Rhizoma (CR),Notoginseng Radix et Rhizoma (NRR), Atractylodis Macrocephalae Rhizoma (AMR), Cirsii Japonici Herba Radix (CJHR), Citri Sarcodactylis Fructus (CSF) and Eucommiae Cortex (EC)] were provided by Zhixin Chinese Herbal Medicine Co., Ltd. (Guangzhou, China) [10]. FTZ was gifted by GUANGDONG METABOLIC DISEASES RESEARCH CENTER OF INTEGRATED CHINESE AND WESTERN MEDICINE (Guangzhou, China). The quality analysis of FTZ extract was performed by HPLC fingerprint method as previously reported [11]. A voucher specimen was deposited in the Institute of Chinese Medicine of Guangdong Pharmaceutical University. Atorvastatin as the control drug was provided by Pfizer (USA).

### Animals

Forty male New Zealand rabbits (1.8–2.2 kg) were supplied by Guangdong Medicinal Laboratory Animal Center, Guangzhou, China (Certification: SYXK Guangdong 2012–0125). All animal procedures were performed in accordance with the protocols approved by the Committee for Supervision of Animal Experiments of Guangdong Pharmaceutical University (Guangzhou, China). After one week of acclimatization, forty rabbits were randomized into five groups (*n* = 8): blank group, restenosis group, FTZ group, atorvastatin group and FTZ + atorvastatin group.

Except for blank group receiving shame operation, the other four groups were given the following operations: the animals were anesthetized with auricular vein injection of pentobarbital sodium (1 mg/kg) and supine position fixation. Then 6F (1F = 0.33 mm) artery sheath tubes (Terumo Corporation, Japan) were introduced by guide wires through the femoral arteries and advanced into the abdominal arteries under the digital subtraction X-ray machine (Artis zee III ceiling, Siemens, Germany). The Sprinter 3.0 mm × 20 mm balloons (Metronic, USA) were inflated with 303.975 kPa and then moved up and down through the abdominal aortas three times to ensure adequate damage of the endothelium. The wounds were adequately pressurized and enswathed. Penicillin sodium was injected inside the thighs for 3 days to avoid infection. Then, the rabbits were fed high fat diet (1.5% cholesterol, 0.5% sodium cholate, 8% lard, 10% egg yolk powder) for 4 weeks to form atherosclerosis. In order to prevent diarrheal, the rabbits were fed yeast tablets 0.2 g/ day.

After 4 weeks of high fat diet, the four groups underwent angiography and rubbing of endothelium by balloon to generate restenosis. Then, the rabbits were fed regular chow for another 4 weeks and given corresponding drugs including FTZ (0.66 mg/kg/day) in FTZ group, atorvastatin (5 mg/kg/day) in atorvastatin group (5 mg/kg/day), and FTZ (0.66 mg/kg/day) plus atorvastatin (5 mg/kg/day) in FTZ + atorvastatin group. At the eighth week, angiographic analyses were performed to verify the effects of drug treatment. Then all animals were sacrificed and abdominal aortas were taken for further pathological analysis. Body weights were measured and food intake was monitored weekly. Meanwhile, 10 ml of arterial blood was taken for biochemical analysis.

### Angiographic analysis of arterial stenosis

The imaging results of each group were analyzed by two-dimensional QCA original workstation from digital subtraction X-ray machine (Artis zee III ceiling, Siemens, Germany). Main measurement indicators: (1) the length of two different angles of the target vessel (take the relative more exposure of vascular lesions); (2) Reference Vascular Diameter: The sum of the proximal diameter and distal diameter of the lesion; (3) % MLD = (1-actual diameter/reference diameter) × 100% (The average sum of the diameter stenosis rates of two different angles); (4) % MLA = (1 - actual stenosis area/reference area) × 100% (The average sum of the area narrow rates of two different angles).

### Biochemical analysis

Blood samples were collected from the abdominal aortas and left at room temperature for coagulation. The serum samples were obtained by centrifugation at 3000 rpm for 15 min at 4 °C, the separated serum was frozen at − 80 °C for further analysis. Serum levels of LDL-C, HDL-C, TC, TG, very low-density lipoprotein cholesterol (VLDL-C) and C-reactive protein (CRP) were analyzed by a blood chemistry analyzer (Hitachi 7108ISE, Japan).

### Elisa

The concentrations of TNF-a, IL-1, IL-6, IL-8, IL-12, ICAM-1 and MCP-1 in serum were measured using commercial ELISA kits (Wuhan Huamei Biological Engineering Co., Ltd., China) according to the manufacturer’s instructions. The optical density of samples was detected using an automated Spectra Max190 microplate reader (Thermo Fisher, USA) at 450 nm.The levels of cytokines were calculated based on a corresponding standard curve.

### Histomorphometric analysis

Abdominal aortic tissues of rabbits were harvested for histopathological examination. The tissue samples were washed with saline and fixed in 4% paraformaldehyde for 48–72 h, dehydrated with graded alcohol, embedded in paraffin and then artery sections (5 μm) were stained with hematoxylin-eosin (HE) and immunohistochemical staining. Morphological changes in the aortas of HE were observed with an optical microscope (Olympus, Japan). The intima (IT), the intima-to-media ratios (IMRS) and medial thickness were calculated using the Image ProPlus 6.0 Analyzer software (Media Cybernetics, Silver Spring, MD, USA). IMRS was used to reflect the intimal hyperplasia of the injured vessels.

### Western blot analysis

Proteins were extracted from injured abdominal arteries and equivalent amounts of protein from each sample were electrophoresed on a 5% discontinuous polyacrylamide minigel, and transferred onto nitrocellulose membranes. The membranes were blocked at 4 °C overnight with 5% non-fat dry milk, then incubated with antibodies for NF-κB (Thermo Fisher, USA), IκB-α, P-IκBα, IKK-α, and P-IKKα/β (all from BIOSS, China) at 4 °C overnight. The membranes were washed and incubated with secondary antibodies for 30 min at room temperature. The immune complexes were visualized using the enhanced chemiluminescence method. Protein levels were quantified by using PhotoShop (version 18.0, Adobe Corporation, USA).

### Statistical analysis

Statistical analyses were conducted using SPSS21.0 software. Results were reported as the mean ± standard deviation and analyzed by analysis of variance (ANOVA) with Tukey’s post hoc test. Differences were considered significant at *P < 0.05*.

## Results

### Successful establishment of restenosis model

**Fig. 1:**
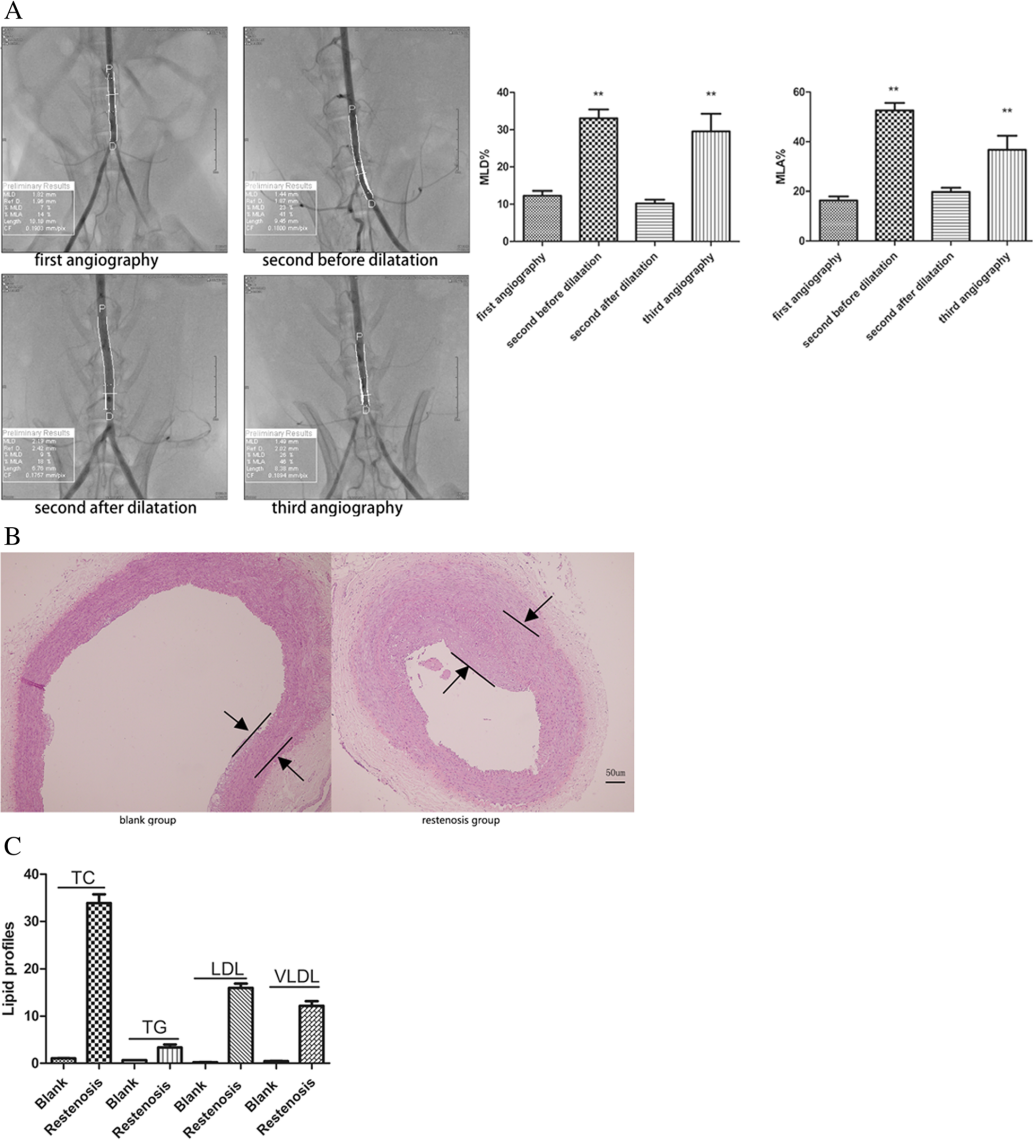
The establishment of the restenosis model. (**a**) Left panels: Angiographic image results in each operation. Right panels: Quantitative analysis of MLD% and MLA% in each operation. **P < 0.01 vs. first angiography. (**b**) HE staining of aortic tissues from blank group and restenosis group. (**c**) Quantitative analysis of serum lipid levels blank group and restenosis group

As shown in Fig. 1a, MLD% and MLA% significantly increased (MLD%:12.2 ± 9.9% vs. 33.0 ± 15.6%, *P < 0.01*; MLA%:16.4 ± 11.2% vs. 52.5 ± 20.0%, *P < 0.01*) in second angiography, compared with the first abdominal aortic angiography. MLD% and MLA% in the restenosis group were significantly increased in the third operation, compared with the second angiographic after balloon dilatation (MLD%:29.5 ± 11.6% vs. 10.2 ± 5.7%, *P < 0.01*; MLA%:36.7 ± 18.7% vs. 19.7 ± 9.7%, *P < 0.01*).

Furthermore, pathological analysis indicated that there was no thickening and lipid deposition, monolayer cells were close to the inner elastic plate, and the middle vascular smooth muscle cells (SMCs) were arranged neatly in the blank group. However, the restenosis group showed severe neointimal hyperplasia and internal elastic plate discontinuity. The intima was thickened, a large number of SMCs were arranged in a disorderly manner, and foam cells were accumulated in the intima (Fig. 1b). Compared with normal diet rabbits, serum TC, TG, LDL-C, and VLDL-C levels were significantly elevated in high fat diet-fed rabbits (Fig. 1c). Taken together, these data indicate successful establishment of model of atherosclerosis and restenosis.

### FTZ reduces serum lipid levels of rabbits

**Fig. 2:**
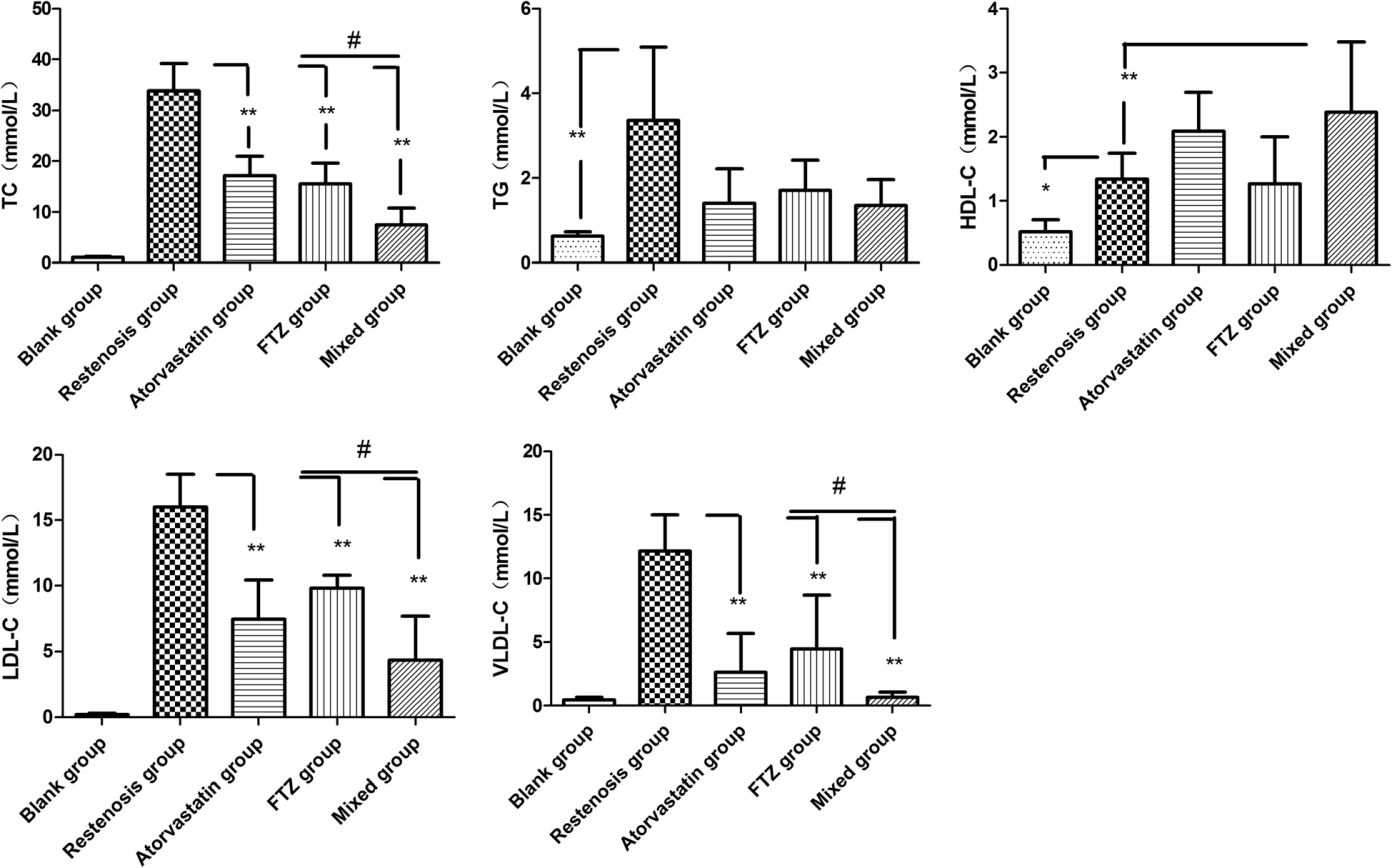
Comparison of serum lipid profiles in each experimental group. Compared with the blank group, most of the lipid profiles were significantly increased in the restenosis group, while the increases were attenuated in drug groups. **P < 0.01 vs. restenosis group, *P < 0.05 vs. restenosis group, #*P* < 0.05 single-used drug group vs. mixed used group

Compared with restenosis group, FTZ and atorvastatin led to significant reduction of serum TC, LDL-C and VLDL-C levels (all *P < 0.0*1) (Fig. 2). In addition, reduced levels of HDL-C in the high fat-fed rabbits were reversed by FTZ and atorvastatin. There were no differences in TC, TG, LDL-C and VLDL-C between FTZ and atorvastatin groups, indicating an equivalent effect of FTZ on lipid profiles compared with atorvastatin. Furthermore, compared with FTZ and atorvastatin group, the levels of TC, LDL-C and VLDL-C were significantly lower in mixed group (*P < 0.05*), and HDL-C was increased, indicating an improved lipid profiles in combination therapy.

### FTZ effectively inhibits vascular restenosis

**Fig. 3:**
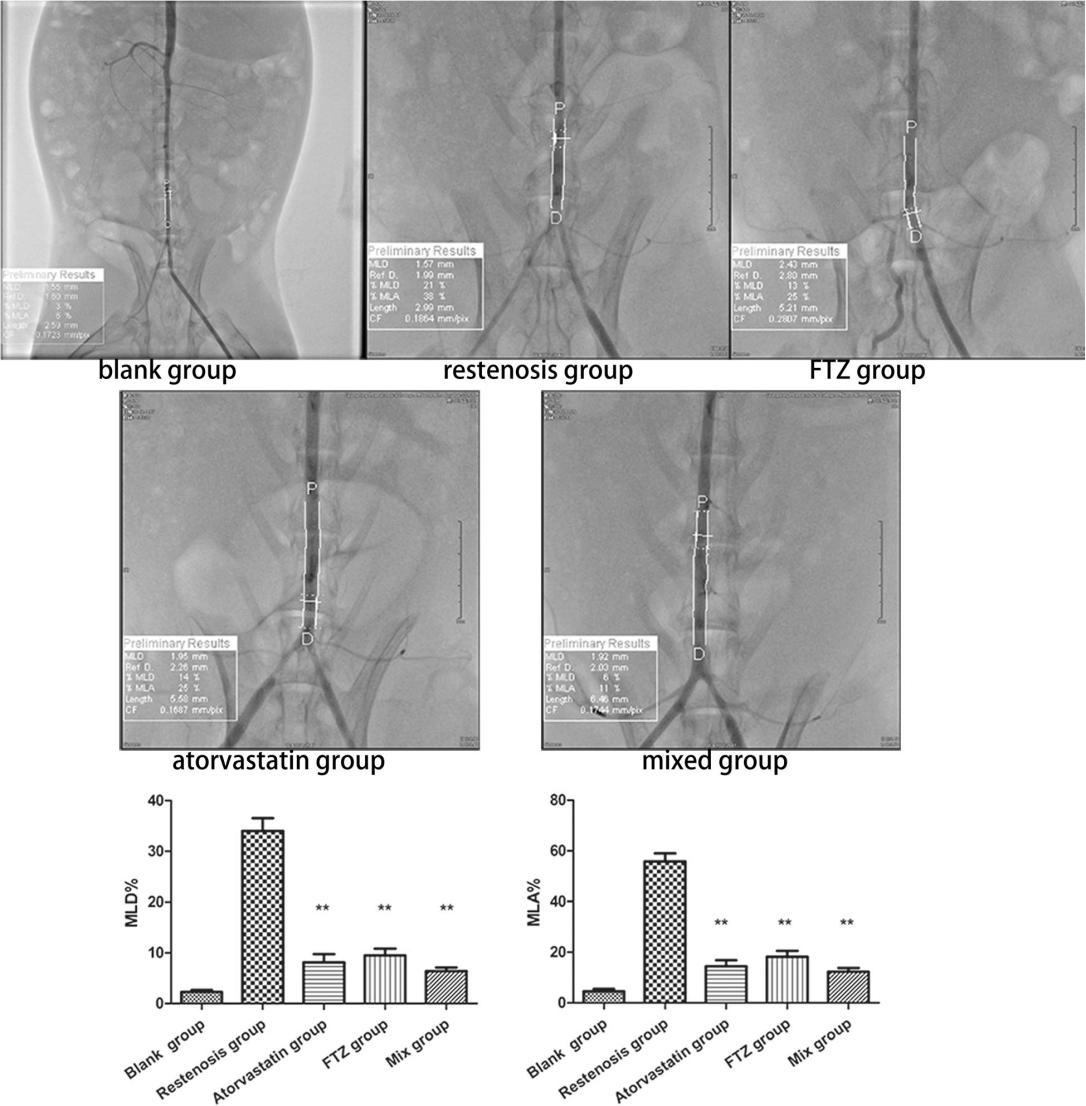
Comparison of angiography results in each group. MLD% and MLA% were significantly increased in the restenosis group, while FTZ and atorvastatin reduced restenosis after PCI. **P < 0.01 vs. restenosis group

Compared with the blank group, MLD% and MLA% were significantly increased in the restenosis group (MLD%:2.2 ± 1.2% vs. 34 ± 7.2%, *P < 0.01*; MLA%:4.6 ± 2.6% vs. 55.7 ± 9.0%, *P < 0.01*). Compared with restenosis group, MLD% and MLA% in FTZ, atorvastatin and mixed groups were significantly decreased (all *P < 0.01*) (Fig. 3).

**Fig. 4:**
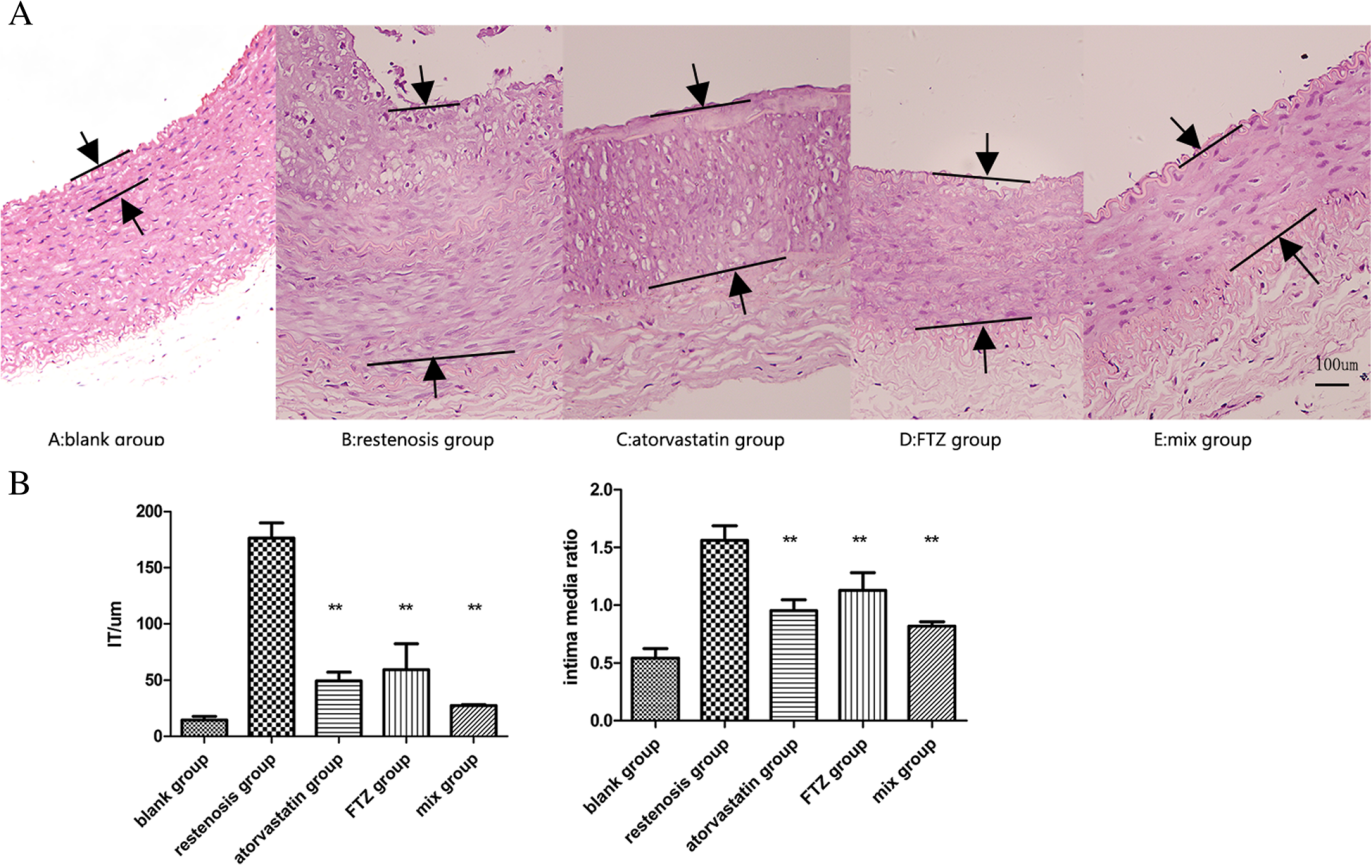
In vivo inhibition of neointimal formation after balloon injuries. (**a**) HE staining of aortic tissues from each group. (**b**) IT and IMRS were significantly thicker in the restenosis group than in blank group, but were reduced in drug groups. **P < 0.01 vs. restenosis group

As shown in Fig. 4, histopathological assessment showed that IT and IMRS in the restenosis group was significantly thicker than that in the blank group (all *P < 0.01*). Compared with the restenosis group, IT and IMRS of drug groups were significantly decreased (all *P < 0.01*). The combination of two drugs were better in decreasing IT and IMRS.

### FTZ reduces serum cytokines levels

Compared with the blank group, serum levels of IL-1, IL-6, IL-8, IL-12, CRP, TNF-α and MCP-1 in restenosis group significantly increased (*P < 0.01*), and serum ICAM-1 level significantly increased too (*P < 0.05*) (Table 1). These data indicate a significant inflammatory response in the restenosis group. However, the levels of pro-inflammatory cytokines IL-1 and IL-8 and CRP TNF-α, MCP-1, and ICAM-1 decreased significantly in treatment groups compared with the restenosis group (all *P < 0.01*). Furthermore, there were no significant differences in the levels of these factors between FTZ and atorvastatin groups, indicating a similar effect in inhibiting inflammatory response. Compared with the single-treatment group, the levels of IL-1 and ICAM-1 were significantly lower in mixed group (*P < 0.05*), which suggested that the combination of two drugs is more effective in inhibiting inflammatory response.

**Table 1:** Serum levels of inflammatory factors in each group

Variable	blank group	restenosis group	atorvastatin group	FTZ group	mixed group
IL-1 (ng/ml)	122.76 ± 11.39	161.96 ± 21.56	94.17 ± 24.53**	94.42 ± 17.77**	75.13 ± 14.01**#
IL-6 (ng/ml)	339.72 ± 7.91**	419.89 ± 43.35	349.64 ± 20.1	358.89 ± 20.92	324.44 ± 33.61
IL-8(ng/ml)	404.26 ± 109.85	994.9 ± 165.16	680.71 ± 53.1**	605.46 ± 16.31**	583.79 ± 154.89**
IL-12(ng/ml)	175.89 ± 11.96**	242.41 ± 33.32	192.57 ± 16.45	194.07 ± 18.76	188.72 ± 10.45
CRP(mg/L)	0.1 ± 0.0x	0.46 ± 0.05	0.13 ± 0.04**	0.16 ± 0.74**	0.13 ± 0.02**
MCP-1 (ng/ml)	214.15 ± 42.67	402.88 ± 41.9	265.92 ± 43.4**	265.48 ± 47.6**	257.99 ± 40.99**
ICAM-1(ng/ml)	121.93 ± 8.16	156.57 ± 20.81	73.37 ± 44.52**	49.99 ± 39.72**	20.31 ± 8.93**#
TNF-a (ng/ml)	12.69 ± 3.85	37.67 ± 20.14	22 ± 3.95**	19.45 ± 2.94**	14.18 ± 3.57**

**P < 0.01 vs. restenosis group, *P < 0.05 vs. restenosis group, #*P < 0.05* means single-used drug group vs. mixed group

### FTZ inhibits NF-κB activation in the aorta

**Fig. 5:**
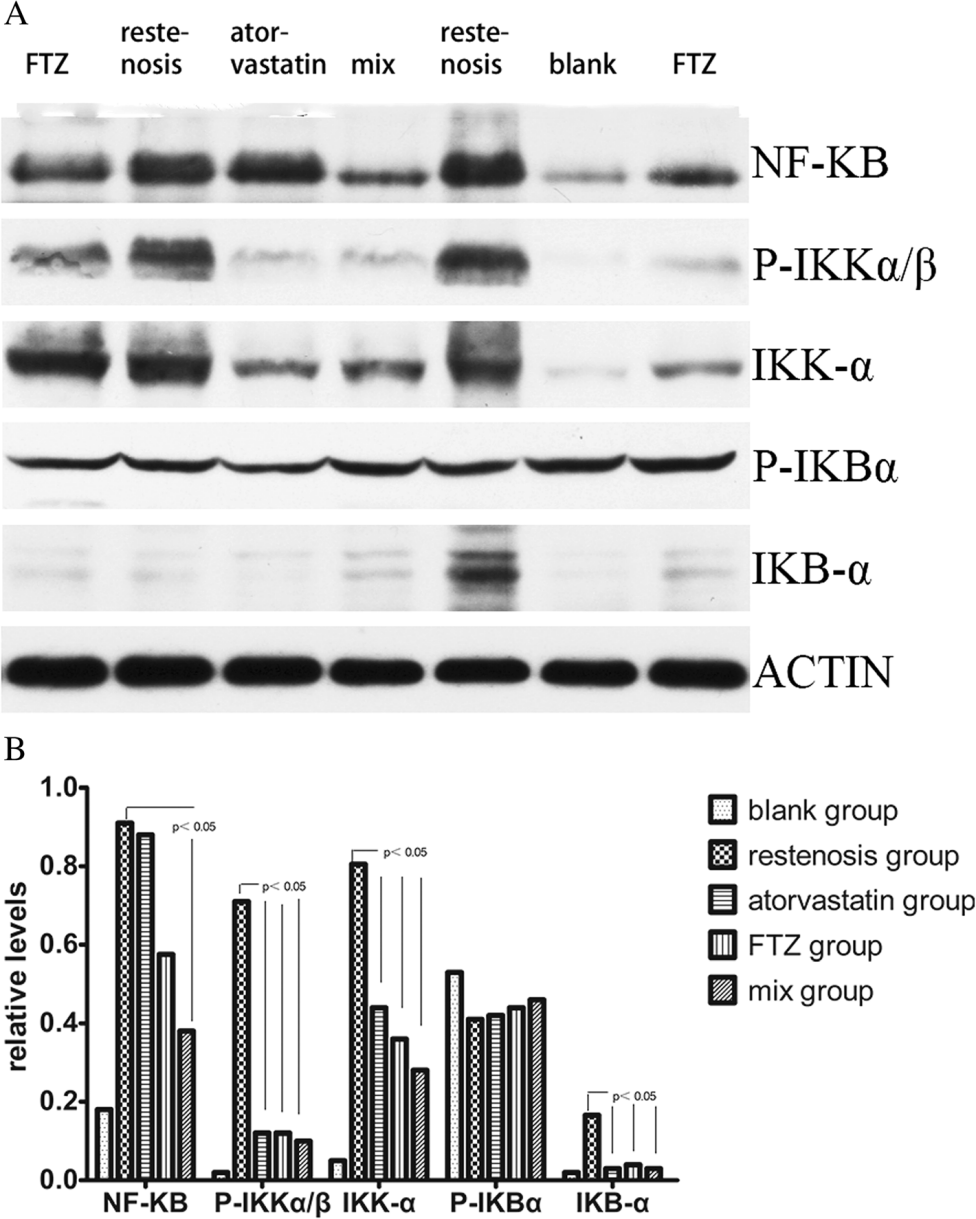
The status of NF-κB pathway activation in abdominal aortas of each group. (**a**) Representative blots showing the levels of NF-κB pathway components in in abdominal aortas of each group. (**b**) Densitometry analysis of the levels of NF-κB pathway components (*n* = 3). **P < 0.01 vs. restenosis group

To understand the mechanism underlying anti-inflammation effects of FTZ, we detected the activation of NF-κB. Western blot analysis showed that the expression of NF-κB, IκB-α, IKKα and P-IKKα/β in abdominal aortas of high fat diet-fed rabbits elevated compared to normal diet-fed rabbits, and administration of FTZ markedly reduced the levels of NF-κB, IκB-α, IKKα, and P-IKKα/β (Fig. 5). Atorvastatin group showed similar effects to inhibit the activation of NF-κB. These results suggest that FTZ plays an anti-inflammatory role by reducing the expression of NF-κB in abdominal aortas.

## Discussion

In this study, a simple, safe, and reproducible model of injury-induced vascular restenosis was established through percutaneous artery puncture. Using this model, we found that FTZ treatment significantly improved vascular restenosis with anti-inflammatory effects in the abdominal arteries.

Hyperlipidemia plays an important role in restenosis. Previous studies have shown that hyperlipidemia could induce inflammation, leading to the formation of restenosis [12]. Lipid-lowering therapy, including statins, was recommended in current PCI guideline. In this study, all rabbits treated with FTZ had significantly lower levels of TC, TG, LDL-C, and VLDL-C. Furthermore, administration of FTZ significantly decreased the restenosis. These results suggest that FTZ is an effective lipid-lowering agent that helps prevent hyperlipidemia-induced restenosis.

Notably, FTZ reduced serum levels of inflammatory factors such as IL-1, IL-6, IL-8, IL-12, CRP, TNF-a and MCP-1. It is known that infiltration of inflammatory cells occurs shortly after endothelial denudation and monocyte infiltration contributes to restenosis [13]. In addition, IL-1 is produced by activated macrophages within atheromatous plaques, and causes pro-inflammatory process involving SMCs proliferation and recruitment of additional inflammatory cells into the plaque and endothelial walls [14]. ICAM-1 is crucial to endothelial leucocytes interaction, and leucocyte migration at the inflammation site. TNF-a plays an important role in vascular inflammation and increases the expression of adhesion molecules [15]. IL-1 is synthesized in endothelial and adventitial cells after balloon injury, and IL-1 related inflammation may play a role in neointima formation [16, 17]. IL-6 promotes the homing of leukocytes into atherosclerotic plaques [18]. High levels of IL-8 were found in atherosclerotic plaques rich in macrophages [19]. Collectively, we postulate that the beneficial effects of FTZ in preventing restenosis might be mediated by inhibiting inflammatory responses induced by hyperlipidemia.

NF-κB plays a crucial role in the coordinated transactivation of cytokines and adhesion molecules, and aberrant activation of NF-κB is implicated in a wide range of human diseases, including inflammation, atherosclerosis and vascular diseases [20]. The inhibiting of NF-κB pathway could reduce neointima formation following vascular injury [21]. Activation of NF-κB can promote the proliferation of vascular smooth muscle cells by regulating the expression of VCAM-1, MCP-1 and TNF-α [22]. Expression of NF-κB was detected in the nuclei within human atherosclerotic lesions [23]. Our results showed that FTZ significantly reduced NF-κB activation, and inhibited the levels of downstream factors of NF-κB, such as IL-1, IL-6, TNF-α and MCP-1, indicating that FTZ targets NF-κB pathway to ameliorate inflammatory cascade in the vessels.

In this study, FTZ showed similar beneficial effects to atorvastatin on restenosis. Previous studies have found that atorvastatin could reduce the inflammation within the atherosclerotic lesion through the inhibition of NF-κB activity and chemokine expression [24]. The effects of atorvastatin on atherosclerosis may be achieved by the inhibition of the expression of TLR4 and NF-κB p65 [25]. Our experimental data showed similar outcomes of FTZ and atorvastatin treatments. In addition, a combination of atorvastatin and FTZ showed better results than treatment with either drug alone.

There are several limitations in our study. First, we used a balloon injury animal model, which is somewhat different from clinical balloon angioplasty procedures performed in patients. Second, the rabbit responses were solely based upon their reactions to injuries whereas mixed pathological inflammation processes and oxidative stress interactions were shown in human responses to vasculature system under an atherosclerotic milieu. Therefore, further clinical research should be performed to evaluate the effects of FTZ on cardiovascular diseases.

## Conclusions

We established a novel vascular restenosis model through percutaneous puncturing of rabbit arteries with balloon injuries and feeding with high fat diet. Based on this model we demonstrated that FTZ significantly improved lipid profiles and downregulated inflammatory factors to inhibit restenosis probably via the inhibition of NF-κB pathway. FTZ may be used as a medication for the treatment of restenosis in clinical practice.

## Abbreviations

CRP: C-reactive protein
FTZ: Fufang-Zhenzhu Tiaozhi Capsule
ICAM-1: Intercellular adhesion molecule-1
IL-1: Interleukin-1
IL-12: Interleukin-12
IL-6: Interleukin-6
IL-8: Interleukin-8
MCP-1: Monocyte chemoattractant protein-1
PCI: Percutaneous coronary intervention
TNF-α: Tumor necrosis factor-alpha
